# Association of Lifestyle and Body Composition on Risk Factors of Cardiometabolic Diseases and Biomarkers in Female Adolescents

**DOI:** 10.1155/2020/9170640

**Published:** 2020-07-09

**Authors:** Valter Paulo Neves Miranda, Paulo Roberto dos Santos Amorim, Ronaldo Rocha Bastos, Karina Lúcia Ribeiro Canabrava, Márcio Vidigal Miranda Júnior, Fernanda Rocha Faria, Sylvia do Carmo Castro Franceschini, Maria do Carmo Gouveia Peluzio, Silvia Eloiza Priore

**Affiliations:** ^1^Department of Physical Education and Department of Nutrition and Health, Federal University of Viçosa, Minas Gerais Postal Code: 36570-900, Brazil; ^2^Department of Physical Education, Federal University of Viçosa, Minas Gerais Postal Code: 36570-900, Brazil; ^3^Department of Statistics-ICE, Federal University of Juiz de Fora, Juiz de Fora-MG, Brazil CEP: 36036-330; ^4^Federal Technology Center of Minas Gerais-Contagem, Minas Gerais, Brazil Postal Code: 36700-000; ^5^School of Physical Education, Physiotherapy and Occupational Therapy, Federal University of Minas Gerais, Belo Horizonte, Minas Gerais, Brazil Postal Code: 31270-901; ^6^Department of Nutrition and Health, Federal University of Viçosa, Minas Gerais, Brazil Postal Code: 36570-900

## Abstract

**Background:**

Female adolescents are considered a risk group for cardiometabolic disease due to their lifestyle (LS).

**Objective:**

To evaluate the association between LS classes and body composition groups with cardiometabolic disease risk factors and pro- and anti-inflammatory biomarkers in female adolescents.

**Methods:**

This cross-sectional study was carried out with female adolescents aged 14 to 19 years, from Viçosa-MG, Brazil. Latent class analysis assessed LS classes. Kinanthropometric measurements were taken together with the body fat percentage (BF%), being analyzed by the Dual Energy X-ray Absorptiometry (DEXA) equipment. Blood pressure and biochemical parameters were analyzed in the Health Division of the Federal University of Viçosa. The pro- and anti-inflammatory biomarkers were analyzed using Luminex technology. Associations with biomarkers were estimated by multiple linear regression.

**Results:**

405 female adolescents were evaluated. The majority, 82.57%, 72.90%, and 65.31%, were classified as inactive by the number of steps, with high screen and cell phone time, respectively. Furthermore, 41.55% did meet the minimum of 60 minutes of moderate-to-vigorous physical activity (MVPA) and 54.69% had high values of BF% (DEXA). The “Sedentary & Inactive LS” class together with the high levels of weight and BF% were associated with increased levels of blood pressure, lipid profile, and uric acid. It was also found that “Inactive & Sedentary LS”, high BF%, insulin resistance, and ultra-sensitive C-reactive protein were associated with the concentrations of proinflammatory biomarkers of tumor necrosis factor-*α*, interleukin-6, and leptin.

**Conclusion:**

We concluded that female adolescents with overweight/obese and high BF% presented higher values of anthropometric indicators, levels of blood pressure, concentration of uric acid and hs-CRP, and lower concentration of HDL. Inactive and Sedentary lifestyle of these girls, along with excess body fat, insulin resistance, and higher concentrations of hs-CRP were associated with the higher concentration proinflammatory markers.

## 1. Introduction

Adolescence is one of the critical moments in life when most behaviors related to lifestyle (LS) are established and can exert influence in adulthood [1]. In this sense, the promotion of a healthy LS among adolescents should be the target of interventions, since several of the modifiable behavioral risk factors, such as physical inactivity, excessive sedentary behavior, inadequate diet, and alcohol and tobacco use are associated with the occurrence of chronic noncommunicable diseases [2, 3, 4].

A high prevalence of unhealthy behaviors is observed during adolescence. Worldwide, about 81.0% of the adolescents aged 11 to 17 years do not meet the minimum recommendation of 60 minutes of moderate-to-vigorous physical activity (MVPA), with this percentage being higher among girls (84.7%) compared to boys (77.6%) [5]. In Brazil, the prevalence of physical inactivity among adolescents is 54.7% (70.7% among females and 38.0% among males) [6]. Regarding sedentary behavior, currently, Canadian adolescents spend, approximately, 9 hours/day of total sedentary time measured by accelerometer [7]. These data contradict the recommendations of the American Academy of Pediatrics, according to which the time in front of the television, video game, or computer, called screen time (ST), should be limited to two hours a day [8]. In Brazil, the last National Survey of School Health (PeNSE), carried out in 2015, showed an excessive ST in 53.8% of the students aged between 13 and 17 years [9].

Such risk behaviors can induce to overweight, mainly due to the imbalance between high consumption of high-calorie diet and low total daily energy expenditure [10]. This positive energy balance can directly contribute to the excess of body fat percentage (BF%) and, consequently, to an increase in blood pressure [11, 12], triglycerides, free fatty acids [13], leptin production [14, 15], dyslipidemias, hyperuricemia [16, 17], and insulin resistance [17, 18].

The metabolic complications mentioned can activate the release of proinflammatory cytokines, such as interleukin-6 (IL-6) and tumor necrosis factor-*α* (TNF-*α*) [19, 20]. IL-6 and TNF-*α* stimulate the production of C-reactive protein (CRP) by the liver [14, 20, 21] and, together, trigger the process of subclinical inflammation, which can result in the development of cardiovascular diseases [19, 22].

Therefore, since female adolescents are more physically inactive and sedentary than males [23], a multivariate analysis of LS, along with different kinanthropometric measures and body composition data of these adolescents may show an association with the cardiometabolic diseases risk factors and with the process of subclinical inflammation [24, 25]. The present study aimed at assessing the association between LS classes and body composition groups with cardiometabolic disease risk factors and pro- and anti-inflammatory biomarkers in female adolescents.

## 2. Material and Methods

This cross-sectional study was carried out with female adolescents ranging from 14 to 19 years of age, enrolled at public schools in Viçosa-MG, Brazil, and living in the same city. The protocols and measures were according to Miranda et al. [24].

The study was approved by the Committee for Ethics in Research with Human Beings of the Federal University of Viçosa (FUV) and filed on the Brazil Platform under the reference number 30752114.0.0000.5153, decision 700.976/2014. The present project followed the rules set by the Declaration of Helsinki and by the Brazilian National Health Council Resolution 466/12. Each volunteer only took part in the project after turning in the Assent Form and the Informed Consent Form, signed, respectively, by themselves and by their parents or legal guardians. Participants 18 or 19 years old just turn in the Informed Consent Form, assigned by them. Both forms contained detailed descriptions of the project and assured the safety, confidentiality, and privacy of the collected information.

In 2014, there were 1.657 adolescents in this age range regularly enrolled in the schools of this city. A cluster sampling plan was used, proportional to the number of adolescents enrolled in the two public schools (clusters) with the largest number of students. This sampling procedure is a probabilistic technique in which sample units are clusters of elements (adolescents). Thus, all eligible students enrolled in the selected schools were invited to participate in the study. A design effect estimated at 1.4 was introduced to correct the variance of parameter estimates, accounting for intracluster correlations. A value greater than one for the design effect indicates that the sample design used is less efficient than simple random sampling.

From this information, the sample size was calculated using the StatCalc software program EpiInfoTM, version 7.2.0.1 (Georgia, United States, 2012). To calculate it, we set the population size at 1.657, confidence level of 95%, the prevalence of outcomes at 22.6% of adolescents with overweight and obesity [26], and maximum error of 5%. The estimated minimum sample size was 324 individuals. To this number, we added 20% to cover for possible losses, making up a total of 389 adolescents. First, all female adolescents were invited to participate in the study. Then, the adolescents were randomly selected to start the assessments. In the end, a total of 405 adolescents participated in all evaluations, with some missing data.

The following inclusion criteria were adopted: being between 14 and 19 years old, having started menstrual function (menarche), voluntarily accepting to participate in the project (or having signed permission from the parents or legal guardian, if under 18), having no previous diagnosis of any type of chronic or infectious disease, not being in the use of any type of antibiotic or other types of medicine that interferes with the metabolism, not participating in other research involving body composition assessment or nutritional status control, not being in the use of probiotic or prebiotic supplements, and having taken no antibiotics for the past three months.

### 2.1. Data Collection Procedures

Data collection procedures started in June 2014 and finished in December 2015. The first stage took place in the schools after consulting with and getting approval from the principal. The students received an explanation about the procedures along with the forms.

In the second stage, all the body composition measurements and biochemical tests were performed. Besides that, 500 microliters of blood serum were separated to be used in the evaluation of the inflammatory markers.

The third stage included an explanation and preparation of the instruments pedometers and 24-hour recall of Physical Activity Level that were used to assess the lifestyle (LS) of female adolescents for eight consecutive days.

### 2.2. Sociodemographic Information

Sociodemographic information and indicators of alcohol and tobacco use were collected by members of the research project. From the date of birth, ages were calculated through the WHO AnthroPlus software and categorized as middle (from 14 to 16) and late adolescence (17 to 19) [27]. Socioeconomic classification was based on the questionnaire proposed by the Brazilian Association of Survey Companies [28].

### 2.3. Body Composition Assessment

A previously trained female researcher performed all the anthropometric measurements. Weight was measured on an electronic digital scale (Kratos®, Campinas-SP, Brazil), and height was measured with a portable stadiometer (Alturexata®, Belo Horizonte, Brazil). Subsequently, the Body Mass Index (BMI) was calculated by Z-score in the WHO AnthroPlus software. The BMI classification was based on the cut-off points proposed by De Onis et al. [29].

The participants went through a 12-hour fasting. Total BF% was evaluated by a Dual-Energy X-ray Absorptiometry (DEX) device (Lunar Prodigy Advance DEX System-analysis version: 13.31, GE Healthcare, Madison, WI, USA). BF% was assessed according to the cut-off points proposed by Williams et al. [30]. BF% above 30.0% was considered high. Girls who participated in the study were grouped into three different groups according to their BMI classification and BF%: Group 1 (G1–control group), Low weight/Eutrophic (LW-EUT) & adequate BF%; Group 2 (G2), EUT and high BF%; and Group 3 (G3), Overweight/Obesity (Ow-OB) & high BF%.

To measure the waist circumference (WC), we used a 2-meter, flexible, and inelastic measuring tape (Cardiomed®, São Luis, MA, Brazil), divided into centimeters and millimeters. Measurements started at the midpoint between the lower margin of the last rib and the iliac crest, on the horizontal plane. For WC classification, the 90th percentile (90th P) was considered as standard [31]. The waist to height ratio (WtHR) was obtained by dividing the waist circumference (cm) by the height (cm).

The neck circumference (NC) was measured at the midpoint of the neck height. The cut-off point used for NC classification was 34.1 cm as observed by Silva et al. [32] in Brazilian adolescents.

### 2.4. Risk Factors for Cardiometabolic Diseases

#### 2.4.1. Biochemical Markers

The biochemical analyses were performed between 07 : 00 and 09 : 00 a.m. by a certified laboratory. Blood samples were collected after a 12-hour fast from an antecubital vein and centrifuged at 2225 × g for 15 minutes at room temperature (2–3 Sigma, Sigma Laborzentrifuzen, Osterodeam Harz, Germany).

First, total cholesterol (TC), high-density lipoprotein (HDL), low-density lipoprotein (LDL), very-low-density lipoprotein (VLDL), and triglycerides concentrations were analyzed. These analyses were done on blood serum after the material was centrifuged in an Excelsa centrifuge, model 206 BL for 10 minutes at 3,500 g. The enzymatic colorimetric method was used to measure TC, HDL, and triglycerides with automation by Cobas Mira Plus equipment (Roche Corp.).

The lipid profile was assessed according to the 2017 Brazilian Guidelines for Dyslipidemia and Prevention of Atherosclerosis [33]. TC, triglycerides, and LDL values were considered high when greater than or equal to 150 mg/dL, 100 mg/dL, and 100 mg/dL, respectively. HDL below or equal to 45 mg/dL was considered low.

Fasting glycemia was measured by the enzymatic method of Glucose Oxidase using the Cobas Mira Plus automation device (Roche Corp.) [33].

Fasting insulin was measured by the electrochemiluminescence method and classified according to the Guidelines of the Brazilian Diabetes Society, which considers high fasting plasma insulin higher than 15*μ*U/mL [33].

Fasting glycemia was measured by the enzymatic method of Glucose Oxidase using the Cobas Mira Plus automation device (Roche Corp.) [33].

The mathematical model Homeostasis Model Assessment–Insulin Resistance (HOMA-IR) was used to calculate insulin resistance using insulin and fasting blood glucose measurements, according to the formula: HOMA − IR = [(fasting insulin (*μ*U/mL) x fasting blood glucose [mmol/L])/22.5] [16]. Values of HOMA-IR higher than 3.16 were considered elevated [34].

Uric acid was measured by the enzymatic colorimetric method, with automation by the Cobas Mira Plus equipment (Roche Corp., Indianapolis, United States) [35]. High-sensitive C-reactive protein (hs-CRP) was measured by the Immunoturbidimetry method [36].

#### 2.4.2. Inflammatory Markers

The evaluated inflammatory markers were interleukin-6 (IL-6), tumor necrosis factor-alpha (TNF-*α*), leptin, and interleukin-10 (IL-10). For this, 500 *μ*L of serum was separated from each blood sample and stored in an ultra-freezer at -80° C until the day of evaluation. These markers were dosed by the Multiplex system-Luminex ™ xMAP technology (Multi Analyte Profile, x = cytokines) using the HMHEMAG-34 K kit (IL-6, TNF-*α*, and leptin).

The MILLIPLEX™ kits were purchased from Merck Millipore Corporation (Merck KGaA, Darmstadt, Germany), and the analyses were performed in a specialized laboratory.

### 2.5. Lifestyle Assessment

LS was considered a latent variable, that is, not directly observable, and was evaluated by Latent Class Analysis (LCA) [37]. With the information from the manifested variables, we fit a statistical model that allowed estimating the probability of a given individual belonging to each of the latent variable categories [38].

In this study, the manifest variables were moderate-to-vigorous physical activity (MVPA), number of steps, sedentary behavior, number of meals, and alcohol and tobacco use. All these variables were evaluated during eight consecutive days. The first day of evaluation was discarded to minimize the Hawthorne effect, which consists of changing the behavior to fulfill the expectations of the study [39].

The PA was evaluated by the Digiwalker SW 200 pedometer (Yamax, Japan), using a cut-off value of 11,700 to determine if the number of steps could be considered an active or inactive behavior [40]. The 24 h recall (R24h) complemented this evaluation [41]. The pedometer recorded participant's scored activities performed in 24 hours (every 15 minutes); MVPA was defined as activities with a metabolic equivalent (MET) equal to or above 3. The MET corresponds to the metabolic-rate multiple needed for an individual to remain at rest. For this study, the adequate average daily time for MVPA considered was at least 60 minutes [42].

Sedentary behavior was assessed by screen time (ST), cell phone screen time (CT), and sitting time during weekdays and weekends. ST and CT were measured according to the questionnaire proposed by Miranda et al. [24], which evaluates the time spent per day in front of a television, computer, video game, and tablets. CT was analyzed separately from the other electronic devices. Both analyses classified the activities as high when the average time in the evaluated days was greater than or equal to 120 minutes per day, which is the cutoff proposed by the [43].

We analyzed the sitting time during weekdays and weekends according to section four of the International Physical Activity Questionnaire (IPAQ) [44]. The weighted average of both data allowed us to estimate the sitting time of both weekdays and weekends. The 75^th^ percentile (75^th^ P) was used as the reference value for sitting time classification due to the lack of a specific cutoff point. The 75^th^ P for all days assessed was 585 minutes.

The number of daily meals was recorded based on breakfast, collation, lunch, afternoon snack, dinner (or snack), and supper. The mean value during the seven days was calculated and later categorized by the 50th percentile (*P*_50_ = 4.0). Values lower than 50th percentile were considered a relatively small number of meals.

Alcohol and tobacco use were observed by two short modules of the Global School-Based Student Health Survey (GSHS) [45]. The answer option represented by the letter “a” for all questions showed that the teenager had never used any type of alcohol and tobacco. The other responses were coded with a numerical score of increasing order to be able to quantify the consumption of alcoholic beverages and the exposure to tobacco.

### 2.6. Statistical Analysis

Statistical analyses were performed using the Statistical Package for the Social Sciences (SPSS) for Windows, version 20.0 (IBM Corporation®, New York, United States). We completed the statistical analysis in the STATA software, version 13.0 (StataCorp LP®, Texas, United States), and the free statistical software R (R Development Core Team, 2014), version 3.2.2 (“Fire Safety”). The level of significance was set at 5%.

The Kolmogorov-Smirnov test and values for the statistics of skewness and kurtosis evidenced nonnormal data. Therefore, results were presented as medians and interquartile ranges (IQR).

Latent class analysis (LCA) was used for modeling the “LS” variable, having been conducted in the poLCA package (Polytomous Variable Latent Class Analysis) available in the library of the R statistical software (R Development Core Team, 2014). The best-fit model with three latent classes has already been presented by Miranda et al. (2019–BMC Public Health). The manifest variables included MVPA, number of steps, ST, number of meals, and total sitting time, with consume of alcohol used as covariate (AIC = 1952.33, BIC = 2024.22, *χ*^2^ = 20.06 (df. = 12, *p* value =0.066), and entropy = 0.79) [24].

The Mann-Whitney and Kruskal-Wallis tests were used to test differences between two or more groups, respectively. The Bonferroni post-hoc test was used to verify differences between pairs of groups. This correction was calculated by dividing the value of total significance (*α* = 0.05) adopted by the number of comparisons between three latent classes and also between the three groups formed according to BMI and BF%. Thus, the value of the Bonferroni correction was equal to 0.0166. Effect sizes were calculated for the differences among groups. For this, the calculator for the Wilcoxon signed-rank test, Mann-Whitney-U test, or Kruskal-Wallis-H test to calculate *η*2 [46]. The effect sizes were classified according to the cut-off points suggested by [47].

Initially, simple linear regression analysis considered the proinflammatory cytokines IL-6, TNF-*α*, and leptin as dependent variables. The values of these markers were log-transformed to meet the assumptions of normal data distribution required in the regression analysis, thus resulting in better fitting models. Only the anti-inflammatory biomarker IL-10 did not show adequate adjustment after transformation, so it was not analyzed in the linear regression models.

VLS, BF%, blood pressure, and biochemical parameters were included in the model as independent variables. In the regression analyzes (simple and multiple), the latent classes 1 (“Active & Sedentary” LS) and 2 (“Inactive & Non-sedentary” LS) were collapsed and considered as the reference class, for class 3 (“Inactive & Sedentary” LS).

A multiple linear regression model was fit right after simple linear regression. In the final model, independent variables were included if a *p* value equal to or less than 0.200 was obtained in the simple linear regression. The backward method was used to reach the final model, with the variables in the order of least significance (highest *p* value) being removed one by one from the model. The procedure was repeated until all the variables included in the model had statistical significance (*p* < 0.05). The interpretation of the estimated coefficients was performed by the exp*β* coefficient.

The significance of the final model was assessed by the F test of the analysis of variance and the quality of the adjustment by the coefficient of determination (adjusted *R*^2^). The residuals were evaluated according to the assumptions of normality, homoscedasticity, linearity, and independence. In addition, multicollinearity was checked using the VIF (Variance Inflation Factor) test among the variables included in the model.

## 3. Results

The average age of the 405 female adolescents evaluated was 15.92 (±1.27) years old, 259 (69%) of whom were in the middle stage of adolescence (14 to 16 years old). Most walked less than 11,700 steps per day (82.57%) and 41.55% reported doing less than 60 minutes of MVPA daily.

The evaluation of sedentary behavior showed that the ST and cell phone time were above 120 minutes/day in 72.90% and 65.31%, respectively. Approximately 50% reported having less than 4 meals usually (Median = 4, IQR = 3.4 − 4.57). Regarding to the alcohol and tobacco use, 56.3% and 62.5% reported having already consumed alcohol and were exposed to some form of tobacco, respectively.

**Figure 1:**
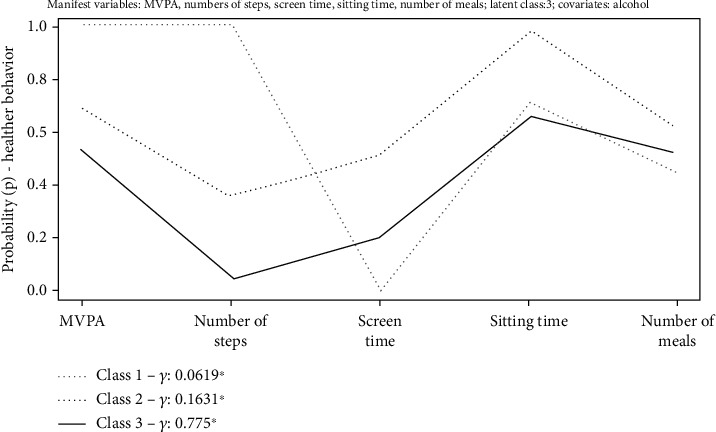
Latent Class Analysis model of female adolescents' lifestyle (LS). ^∗^Prevalence (*γ*) of latent class. *ρ*: item-response probability. Class 1: Active & Sedentary LS (*γ* = 6.19%); Class 2: Inactive & Non-Sedentary LS (*γ* = 16.31%); Class 3: Inactive & Sedentary LS (*γ* = 77.5%). MVPA: Moderate to Vigorous Physical Activity.

The three latent classes were labeled as “Active & Sedentary LS” (class 1-*γ* = 6.15%), “Inactive & Non-sedentary LS” (class 2-*γ* = 16.31%), and “Inactive & Sedentary LS” (class 3-*γ* = 77.5%) (16). Female adolescents that had “never consumed alcohol” were 2.26 times more likely (log OR = 0.8174; *p* = 0.033) to belong to class 3 (“Active & Sedentary” LS) than to class 1 (“Inactive & Sedentary” LS). There wasn't an association between class 2 and class 1 (*p* = 0.781) (16). More details about the three classes can be seen elsewhere Miranda et al. [24] (see Figure 1).

Table 1 shows the absolute and relative frequencies of biochemical and body composition variables.

The evaluation of inflammatory biomarkers showed that IL-6, TNF-*α*, leptin, and IL-10 presented, respectively, median values of concentration equals to 1.95 pg/mL (1.27-2.87), 2.05 pg/mL (1.24-2.8), 4841.5 pg/mL (2818.2-7858.7), and 1.38 pg/mL (1.0-2.07).

**Figure 2:**
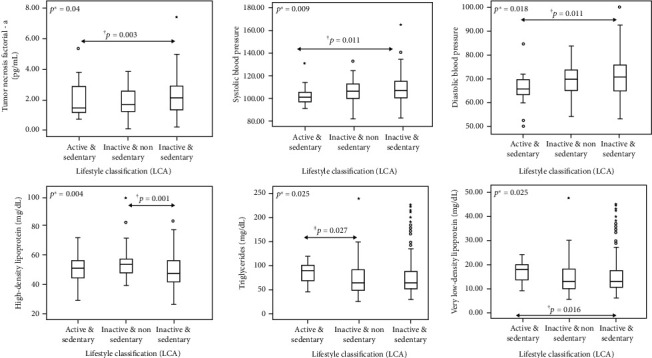
Significant differences on cardiometabolic disease risk factors and inflammatory markers among the latent classes that represent the adolescents' lifestyle. ^∗^Significant *p* values (*p* < 0.05) of Kruskal-Wallis test; ^†^significant *p* values of Mann-Whitney test after Bonferroni correction (≤0.0166) between groups 1 and 3; ^‡^nonsignificant *p* values of Mann-Whitney after Bonferroni correction (>0.0166) between classes 1 and 2. LCA: Latent Class Analysis.

Among the three latent classes, there was variation (*p* value < 0.05) between the values of SBP, DBP, HDL, VLDL, triglycerides, and TNF-*α* (Supplementary material 1). “Inactive & Sedentary LS” class (class 3) showed higher values of SBP, DBP, and TNF-*α*, as well as lower values of HDL. On the other hand, the “Active & Sedentary LS” class (class 1) showed higher values of VLDL and triglycerides. These results are highlighted in Figure 2.

In addition, we found a difference in the values of biochemical tests and inflammatory biomarkers among the groups of body composition according to the BMI and the BF% (Table 2). Female adolescents classified as “Ow-OB & High BF%” group (G3) had higher values of SBP, DBP, LDL, VLDL, triglycerides, glucose, insulin, HOMA-IR, UC, CRP-us, leptin, and lower HDL values in relation to the “LW-EUT & Adequate BF%” group (G1). There was no difference between the “LW-EUT & Adequate BF%” group (G1) and “EUT & High BF% group” (G2) (Table 2).

Simple linear regression analysis showed the independent variables that were significantly associated with the inflammatory markers (Supplementary material 2). The multiple linear regression models found that the behavioral variables, BF%, insulin resistance, and hs-CRP were linearly associated with the inflammatory biomarkers TNF-*α*, IL-6, and leptin (Table 3). Female adolescents belonging to the “Inactive & Sedentary LS” class (class 3) showed an increase of 1.24 (CI95% 1.07–1.45, *p* = 0.005) in the TNF-*α* concentration unit as compared to the “Active & Sedentary LS” (class 2) and “Inactive and Non-Sedentary LS” (class 3) classes collapsed into a baseline class. Still, this model showed that, with each increase in a unit of hs-CRP, there was an increase of 1.36 (CI95% 1.13–1.63, *p* = 0.006) in the TNF-*α* concentration unit.

Only insulin resistance was linearly associated with IL-6. That is, with each increase of one unit of the HOMA-IR index there was an increase of 1.37 (CI95% 1.26–1.49, *p* = 0.026) in the IL-6 concentration unit. Also, BF% and HOMA-IR were linearly associated with leptin. For each increase of one unit in the BF% and HOMA-IR, there was an increase of 1.06 (CI95% 1.05–1.07, *p* = <0.001) and 1.10 (CI95% 1.03–1.18, *p* = <0.001), respectively, in the leptin concentration unit.

## 4. Discussion

This study evaluated the association between lifestyle and body composition with risk factors for cardiometabolic diseases and pro (TNF-*α*, IL6, and Leptin) and anti (IL-10) inflammatory biomarkers in female adolescents. A critical observation from the analysis is that the concentration of inflammatory markers in female adolescents was associated with the Inactive & Sedentary latent class, body fat percentage (BF%), high-sensitivity C-reactive protein (hs-CRP), and insulin resistance.

Firstly, it is important to note that approximately 70% of the female adolescents evaluated had a high screen and cellular time (≥120 minutes), regardless of the level of physical activity. This confirms the difference between physical inactivity and sedentary behavior. Accordingly, the findings of Tremblay et al. [48] clearly indicate that physical inactivity is different from sedentary behavior. Being physically inactive means not meeting any NAF recommendations for a specific population, such as 60 minutes of MVPA for children and adolescents. On the other hand, sedentary behavior (from the Latin word sedere, “to sit”) describes a distinct class of activities that require low levels of energy expenditure in the range of 1.0–1.5 METs (multiples of the basal metabolic rate) and involve sitting during commuting, in the workplace and the domestic environment, and during leisure.

These findings are in consonance with the systematic review study carried out by [49] where biomarker as hs-CRP was correlated with physical activity and subjects' dietary habits. In addition, an association between overweight (assessed by BMI), high BF%, and central fat accumulation in adolescents were also identified by Miranda et al. [50] and Elizondo-Montemayor et al. [21]. In the present study, results showed that the girls from the Inactive & Sedentary LS class displayed the higher levels of blood pressure (SBP and DBP) and TNF-*α* concentration. In addition, they exhibited lower HDL values, i.e., an association with cardiometabolic risk factors and with an inflammatory biomarker. This emphasizes the importance of encouraging the adolescent population to become more physically active and less sedentary.

Other findings related to lifestyle showed that adolescents from the Active & Sedentary LS group displayed higher values of VLDL and triglycerides compared to the adolescents from the Inactive & Non-sedentary lifestyle. The explanation for this difference may be based on the assumption that sedentary activity, such as high screen time, is related to a greater intake of energetic and hypercaloric foods [49].

It is possible to notice the importance of increasing activity level, decreasing sedentary behavior, and adopting healthy and balanced eating habits during adolescence, to prevent the development of inflammatory process, associated with overweight and body fat [2, 49]. In this research, the evaluation of body composition displayed that 22.5% of the adolescents were overweight or obese, in addition to 54% with elevated (above 30%) BF%. Information from the Study of Cardiovascular Risk in Adolescents (ERICA), conducted by Bloch et al. [26], showed that the prevalence of overweight and obesity in Brazilian adolescents between 12 and 17 years old was of 22.6%, similar to the value found in the present study. Data from the National Health and Nutrition Examination Surveys reveal that the obesity prevalence in female adolescents from the United States, aged 12 to 19 years old, is 21% [51].

The overweight or obese adolescents with elevated BF% (G3) displayed higher values of blood pressure, changes in biochemical parameters, and higher concentrations of hs-CRP, when compared to the eutrophic with adequate BF% (G1). In a previous study conducted by our group [50], adolescents from the EUT & High %BF and OW-OB & High BF groups displayed a higher concentration of central, visceral fat, and leptin than the EUT & Adequate %BF group. These results highlight the role of BF% in the prevalence of cardiometabolic risk factors. In addition, it is noted that a significant portion of adolescents already has risk factors associated with metabolic diseases. These findings should not be ignored, since literature confirms that lifestyle (cause) and comorbidities (consequence) consolidated in adolescence tend to persist in adulthood [49].

The female adolescents' high body fat was also associated with the concentrations of uric acid. Other studies verified the association of hyperuricemia with other metabolic disorders, such as obesity, dyslipidemia, arterial hypertension, and metabolic changes [16, 17]. Uric acid is the end product of purine (adenine and guanine) catabolism and is formed mainly in the liver from xanthine, by the action of the xanthine oxidase enzyme [52]. The high concentration of uric acid can affect the bioavailability of endothelial nitric oxide (NO) [53]. Consequently, the absorption of glucose by the skeletal musculature is decreased, thus, contributing to the increase of insulin resistance [16].

The final multiple linear regression model confirmed that the class with the least healthy behavior “Inactive & Sedentary LS” was associated with the inflammatory biomarker TNF-*α*. In turn, TNF-*α* was also associated with hs-CRP (*p* = 0.001). This is regarded as the main acute phase protein synthesized by the liver and is regulated by proinflammatory cytokines, such as IL-6 and TNF-*α* [3, 54]. The increase of CRP concentration occurs in chronic inflammatory situations, such as atherosclerosis, and its levels nearly triple in the presence of risk of peripheral vascular diseases [21, 55].

Martinez-Gomez et al. [56] found in 1025 adolescents of both sexes that those who engaged in vigorous physical activity for a greater amount of time had lower concentrations of CRP. In the present study, adolescents with “Inactive & Sedentary LS” displayed higher concentrations of TNF-*α* than those with “Active & Sedentary LS” and “Inactive & Non-Sedentary LS”. It is known that physical activities favor the release of anti-inflammatory markers by the skeletal musculature [57].

The Il-6 was only related to insulin resistance (IR), which was evaluated through the HOMA-IR index. In turn, leptin concentration was associated to HOMA-IR and also to %BF, regardless of lifestyle, blood pressure, and biochemical parameters. It is known that adipose tissue increases the secretion of proinflammatory cytokines, such as IL-6, TNF-*α*, and leptin, which are closely related to the development of insulin resistance [13].

Insulin resistance and leptin act to reduce food intake and to increase energy expenditure through the action on the hypothalamic neurons, for which they are named “signals of body adiposity” [14]. Excessive weight contributes to hyperleptinemia, a condition in which the leptin receptors are altered or defective at the blood-brain barrier, resulting in a resistance, and ceasing to regulate body weight and appetite [15, 18]. Baseline leptin and insulin concentrations may be positively correlated to insulin-sensitive individuals and both decrease in response to weight loss [21, 22].

The female adolescents classified as having an active or nonsedentary lifestyle and with adequate percentage body fat were less likely to be associated to the risk factors of cardiometabolic diseases and to inflammatory biomarkers. This suggests that engaging in regular, well-guided physical exercise may be a favorable nondrug measure with respect to the state of metabolic disease and subclinical inflammation.

According to Petersen and Pedersen [58], the muscle cell stimulated by physical exercise produces the IL-6 myokine, which prompts an increase in the production of IL-1 and IL-10 anti-inflammatory cytokines that will, in turn, inhibit the production of TNF-*α*. Hence, it is evident that one of the main considerations of the present study is the fact that a more active and less sedentary lifestyle may act indirectly in the inflammatory process.

The cut-off points of the study include an application of a technique recently introduced in the epidemiology of physical activity—LCA—to identify LS classes among adolescents. In addition, important characteristics of lifestyle and body composition were related to the manifestation of different risk factors for cardiometabolic diseases, and five inflammatory biomarkers were investigated in a representative sample of female school-going adolescents in a city in the state of Minas Gerais, Brazil. However, the study has some limitations that have to be considered. Some measures of behavior related to lifestyle were analyzed subjectively and self-reported. Yet, all the questionnaires used are validated methods and a pedometer was also used to measure physical activity. Another factor to be accounted for is the cross-sectional design of the study, which limits inferences about causality.

Further studies are suggested to verify the association of LS classes involving different manifest variables related to cardiometabolic diseases, such as the consumption of fruits and vegetables and sleep duration, so as to provide a better overview of the profile of adolescents. It is also suggested to conduct longitudinal studies to track adherence to behaviors and the prevalence of risk factors at different times of adolescence.

This study can help educators and health professionals in designing more efficient strategies to encourage female adolescents to adopt a more active lifestyle, less sedentary behaviors, with healthy and balanced diets, aiming at controlling excess weight and body fat. These healthy behaviors may prevent the manifestation of risk factors for cardiometabolic diseases and inflammatory markers, whose early onset in adolescence may worsen in adulthood, thus, triggering cardiovascular diseases.

## 5. Conclusion

This study allowed one to conclude that the inactive and sedentary lifestyle of female adolescents, along with excess body fat, insulin resistance, and higher concentrations of high-sensitivity C-reactive protein are associated to the higher concentration of TNF-*α*, IL-6, and leptin. Also, it was verified that girls classified as inactive and sedentary displayed higher levels of blood pressure, lower HDL concentrations, and higher TNF-*α* concentration. The overweight or obese adolescents with high %BF displayed a higher number of altered biochemical parameters, in addition to higher values of uric acid and hs-CRP, not to mention that the high prevalence of girls with high screen time, use of cell-phones, and percentage body fat.

This study can help educators and health professionals in designing more efficient strategies to encourage female adolescents to adopt a more active lifestyle, less sedentary behaviors, with healthy, and balanced diets, aiming at controlling excess weight and body fat. These healthy behaviors may prevent the manifestation of risk factors of cardiovascular diseases and inflammatory markers. These healthy behaviors may prevent the manifestation of risk factors for cardiometabolic diseases and inflammatory markers, whose early onset in adolescence may worsen in adulthood, thus, triggering cardiovascular diseases.

## Figures and Tables

**Table 1:** Absolute and relative frequencies of female adolescents' lifestyle cardiometabolic diseases risk factors.

Variables	Absolute frequency (*n*)	Relative frequency (%)
BMI & BF%^∗^ (DEXA) (*n* = 395)		
LW-EUT & adequate BF% (G1)	179	45.31
EUT & high BF% (G2)	126	31.90
OW-OB & high BF% (G3)	90	22.78
Neck circumference (cm) (*n* = 405)		
Adequate neck circumference	388	95.80
High neck circumference	17	4.20
Blood pressure (mmHg) (*n* = 400)		
Normotensive	332	83
High blood pressure	68	17
Total cholesterol (mg/dL) (*n* = 403)		
Adequate total cholesterol	218	54.09
High total cholesterol	185	45.91
HDL (mg/dL) (*n* = 403)		
Adequate HDL	274	67.99
Low HDL	129	32.01
LDL (mg/dL) (*n* = 403)		
Adequate LDL	317	78.66
High LDL	86	21.34
Triglycerides (mg/dL) (*n* = 403)		
Adequate triglycerides	338	83.87
High triglycerides	65	16.13
Glucose (mg/dL) (*n* = 400)		
Adequate glucose	395	98.70
High glucose	5	1.30
Insulin (mUI/mL) (*n* = 398)		
Adequate insulin	379	95.20
High insulin	19	4.80
HOMA-IR (*n* = 398)		
Adequate HOMA-IR	367	92.20
High HOMA-IR	31	7.80
Uric acid (mg/dL) (*n* = 402)		
Adequate UA	392	97.51
High UA	10	2.49
hs-CRP (mg/dL) (*n* = 401)		
Adequate hs-CRP	350	87.28
Inflammation	51	12.72

^∗^Body composition classification; n: absolute frequency; BMI: body mass index; DEXA: dual-energy X-ray absorptiometry; LW: low weight; EUT: Eutrophy; OW: overweight; OB: obesity; BF%: body fat percentage; LDL: low-density lipoprotein; HDL: high-density lipoprotein; HOMA-IR: homeostasis model assessment–insulin resistance. hs-CRP: high sensitivity C-reactive protein.

**Table 2:** Quantitative values of the cardiometabolic disease risk factors among the groups of body composition.

Quantitative values of CD factors	Group 1 LW-EUT & adequate BF% (*n* = 179)	Group 2 EUT & high BF% (*n* = 131)	Group 3 OW-OB & high BF% (*n* = 95)	*p* values
	Median (P25-P75)	Median (P25-P75)	Median (P25-P75)	
WC (cm)	66.0 (63.0-68.2)^¥†^	71.3 (68.5-74.0)^¥‡^	82.6 (78.7-87.8)^†‡^	<0.001^∗^
WHtR	0.40 (0.39-0.42)^¥†^	0.43 (0.42-0.46)^¥‡^	0.51 (0.48-0.53)^†‡^	<0.001^∗^
NC	29.6 (29.0-30.7)^¥†^	30.5 (29.5-31.2)^¥‡^	32.6 (31.2-33.5)^†‡^	<0.001^∗^
SBP (mmHg)	103.5 (99.0-110.0)^†^	105.5 (100.0-111.3)^‡^	111.2 (105.0 – 120.3)^†‡^	<0.001^∗^
DBP (mmHg)	69.0 (63.5-73.5)^†^	70.0 (65.5-74.5)^‡^	73.7 (67.6-79.9)^†‡^	<0.001^∗^
Total cholesterol (mg/dL)	145.0 (132.0-161.5)	150.0 (132.2-164.0)	150.5 (134.5-173.2)	0.134
HDL (mg/dL)	52.0 (46.0-58.0)^†^	49.0 (42.0-58.0)	46.0 (39.7-54.0)^†^	<0.001^∗^
LDL (mg/dL)	78.8 (64.9-94.6)^†^	84.5 (70.2-96.3)	87.5 (71.9-109.3)^†^	0.018^∗^
VLDL (mg/dL)	12.6 (9.4-16.0)^†^	13.5 (10.6-17.6)	14.1 (10.8-18.8)^†^	0.01^∗^
Triglycerides (mg/dL)	63.0 (47.0-80.0)^†^	67.5 (53.2-88.0)	70.5 (54.0-94.2)^†^	0.01^∗^
Glucose (mg/dL)	85.0 (80.0-89.0)†	85.0 (80.0-88.0)	87.0 (82.0-91.0)^†^	0.032^∗^
Insulin (mUI/mL)	5.8 (4.6-7.7)^†^	6.6 (4.8-8.6)^‡^	9.1 (6.3-12.9)^†‡^	<0.01^∗^
HOMA-IR	1.3 (1.0-1.7)^†^	1.5 (1.0-1.9)^‡^	2.0 (1.3-3.1)^†‡^	<0.001^∗^
Uric acid (mg/dL)	3.4 (2.9-3.9)^†^	3.6 (3.0-4.2)^‡^	3.9 (3.5-4.9)^†‡^	<0.001^∗^
hs-CRP (mg/dL)	0.04 (0.02-0.10)^†^	0.07 (0.03-0.17)	0.10 (0.04-0.26)^†^	<0.001^∗^
IL-6 (pg/mL)	1.9 (1.2-2.8)	1.8 (1.3-2.8)	2.2 (1.3-3.0)	0.434
TNF-*α* (pg/mL)	1.8 (1.2-2.7)	2.2 (1.2-2.8)	2.1 (1.4-2.8)	0.148
Leptin (pg/mL)	3207.0 (2144.0-4930.0)^†^	5944.0 (3800.0-7794.5)^‡^	9521.0 (6505.7-14175.2)^†‡^	<0.001^∗^
IL-10 (pg/mL)	1.36 (0.9-2.0)	1.4 (1.0-2.3)	1.4 (1.0-2.1)	0.295

^∗^Significant *p* values (*p* < 0.005) of Kruskal-Wallis test; ^¥^significant *p* values of Mann-Whitney test after Bonferroni correction (≤0.0166) between groups 1 and 2; ^†^significant *p* values of Mann-Whitney test after Bonferroni correction (≤0.0166) between groups 1 and 3; ^‡^significant *p* values of Mann-Whitney test after Bonferroni correction (>0.0166) between groups 2 and 3. LW: low weight; EUT: Eutrophy; OW: overweight; OB: obesity; BF%: body fat percentage; SBP: systolic blood pressure; WC: waist circumference; WHtR: waist-to-height ratio; NC: neck circumference; DBP: diastolic blood pressure; HDL: high-density lipoprotein; LDL: low-density lipoprotein; VLDL: very-low-density lipoprotein; HOMA-IR: homeostasis model assessment–insulin resistance; hs-CRP: high sensitivity C-reactive Protein; IL-6: interleukin-6; TNF-*α*: tumor necrosis factor-*α*; IL-10: interleukin-10.

**Table 3:** Multiple linear regression model referent to the association among inflammatory markers, lifestyle (LCA model), and cardiometabolic disease risk factors^**‡**^.

	*TNF-α^†^*						
Independent variables	*β*-Coefficient ln (exp)	CI 95% ln (exp)	Standardized *β*-coefficient ln (exp)	*p* value	*R* ^2^	Adjusted *R*^2^	F test
LCA model ^#^Active & sedentary LS + Inactive & non-Sedentary LS	1	—	—		0.044	0.038	*p* < 0.001
Inactive & sedentary LS	0.221 (1.24)	0.069 (1.07)-0.374 (1.45)	0.161 (1.17)	0.005			
hs-CRP	0.31 (1.36)	0.127 (1.13)-0.494 (1.63)	0.187 (1.20)	0.001			

*IL-6* ^†^							
Independent variable	*β*-Coefficient) ln (exp)	CI 95% ln (exp)	Standardized *β*-coefficient^∗^ ln (exp)	*p* value	*R* ^2^	*R* ^2^ adjusted	F test
HOMA-IR	0.319 (1.37)	0.239 (1.26)-0.399 (1.49)	—	0.026	0.013	—	—

*Leptin* ^†^							
Independent variable	*β*-Coefficient ln (exp)	CI 95% ln (exp)	Standardized *β*-coefficient ln (exp)	*p* value	*R* ^2^	*R* ^2^ Adjusted	F test
BF%	0.064 (1.06)	0.057 (1.05)-0.072 (1.07)	0.431 (1.53)	<0.001	0.34	0.335	<0.001
HOMA-IR	0.101 (1.10)	0.036 (1.03)-0.166 (1.18)	0.186 (1.20)	<0.001			

^‡^The method for selecting variables was backward. ^#^Class 1 and class 2 collapsed; ^†^Cardiometabolic markers that showed normal distribution after logarithmic transformation. ^∗^Standardized *β*-coefficient was not used because with IL-6 only one variable showed association. ln: *β*-coefficient values presented for natural logarithm of independent variables; exp:: exp(*β*)-; TNF-*α*: tumor necrosis factor-*α*; IL-6: Interleukin-6; BF%: body fat percentage; hs-CRP: high sensitivity C-reactive protein; HOMA-IR: homeostasis model assessment–insulin resistance.

## Data Availability

The data used to support the findings of this study have been deposited in the Data_VPNM_03-09-20.xls repository and it was included within the supplementary information file(s). Any further information or questions regarding the data provided, please contact the corresponding author by email (Valter Paulo Neves Miranda - vpnmiranda@yahoo.com.br).

## Supplementary Materials

Supplementary materials Supplementary material 1: quantitative values of cardiometabolic disease risk factors and inflammatory markers according to the latent classes. Supplementary material 2: association of inflammatory markers with lifestyle (LCA model) and cardiometabolic disease risk factors.
