# Supplementary material for: Association of Lifestyle and Body Composition on Risk Factors of Cardiometabolic Diseases and Biomarkers in Female Adolescents
Source: Mediators Inflamm. 2020 Jul 9;2020:9170640. doi: 10.1155/2020/9170640 (PMC7368183; doi:10.1155/2020/9170640)
Supplement: Supplementary materials — Supplementary material 1: quantitative values of cardiometabolic disease risk factors and inflammatory markers according to the latent classes. Supplementary material 2: association of inflammatory markers with lifestyle (LCA model) and cardiometabolic disease risk factors. [file 9170640.f1.pdf]

Supplementary material 1 - Quantitative values of cardiometabolic disease risk factors and inflammatory markers according to the latent classes.

Quantitative values CD factors & Inflammatory Markers	Class 1 (γ = 6.19%) Active & Sedentary LS Median (P25-P75)	Class 2 (γ = 16.31%) Inactive & Non-Sedentary LS Median (P25-P75)	Class 3 (γ = 77.5%) Inactive & Sedentary LS Median (P25-P75)	*p-values*
BMI (kg/m²)	21.1 (19.5-24.4)	20.72(19.1-23.3)	21.18 (19.1-24.5)	0.89
BF%	28.3 (24.6-32.2)	29.0 (26.2-34.5)	31.2 (26.2-37.4)	0.25
WHtR	0.41 (0.39-0.44)	0.42 (0.40-0.46)	0.43 (0.40-0.48)	0.707
Neck Circumference	30.0 (29.02-31.0)	30.7 (28.9-31.9)	30.5 (29.2-31.7)	0.34
SBP (mmHg)	105.0 (100.0-110.5)†	105.5 (99.0-112.5)	107.5 (100.0-115.0)†	**0.009***
DBP (mmHg)	70.0 (65.0-75.0)†	69.5 (65.0-73.5)	70.7 (65.0-76.1)†	**0.018***
Total Cholesterol (mg/dL)	148 (141.0-162.5)	151.5 (130.2-164.0)	146.0 (131.0-165.0)	0.657
HDL (mg/dL)	51.0 (42.0-57.1)	54.0 (48.0-58.0)†	48.0 (42.0-57.0)†	**0.004***
LDL (mg/dL)	80.6 (66.5-99.9)	70.0 (68.5-93.4)	82.4 (69.0-96.6)	0.646
VLDL (mg/dL)	18.0 (13.7-20.1)‡	12.6 (9.6-18.0)‡	13.0 (10.3-17.1)	**0.025***
Triglycerides (mg/dL)	90.0 (68.5-100.5)‡	63.0 (48.2-90.2)‡	65.0 (51.7-86.0)	**0.025***
Glucose (mg/dL)	85.0 (81.0-88.0)	85.5 (81.0-90.0)	85.0 (80.2-89.0)	0.777
Insulin (mUI/mL)	6.6 (4.6-8.2)	6.3 (5.0-8.3)	6.8 (51.2-9.2)	0.654
HOMA-IR	1.5 (1.0-1.8)	1.4 (1.0-1.9)	1.5 (1.1-2.0)	0.8
Uric Acid (mg/dL)	3.4 (3.1-4.0)	3.5 (3.0-3.9)	3.6 (3.0-4.3)	0.46
hs-PCR (mg/dL)	0.06 (0.03-0.15)	0.04 (0.02-0.17)	0.07 (0.02-0.17)	0.82
IL-6 (pg/mL)	2.5 (1.3-4.26)	1.9 (1.3-3.4)	2.01 (1.3-2.8)	0.281
TNF-α (pg/mL)	1.4 (1.1-2.8)	1.7 (1.1-2.5)†	2.1 (1.3-2.8)†	**0.04***
Leptin (pg/mL)	6144.0 (2882.8-9851.1)	4974.0 (2708.7- 6515.5)	4988.0 (2974.0-8291.7)	0.362
IL-10 (pg/mL)	1.4 (1.0-2.2)	1.4 (1.0-1.9)	1.4 (1.0-2.2)	0.724

* *significative p*-values (p<0.005) of Kruskal Wallis test; †significative p-values of Mann-Whitney test less or equal than Bonferroni significance correction (≤0.0166). ‡significative p-values of Mann-Whitney test larger than Bonferroni correction (>00166).

BMI: Body Mass Index; BF%: Body Fat percentage; SBP: Systolic Blood Pressure; DBP: Diastolic Blood Pressure; HDL: High Density Lipoprotein; LDL: Low Density Lipoprotein; VLDL: Very Low Density lipoprotein; HOMA-IR: Homeostasis Model Assessment – Insulin Resistance; hs-CRP: high sensitivity C-reactive Protein; IL-6: Interleukin-6; TNF-α: Tumor Necrosis Factor-α; IL-10: Interleukin-10.

Supplementary material 2 – Association of inflammatory markers with lifestyle (LCA model) and cardiometabolic disease risk factors.

Variables	**†TNF-α**				**†IL-6**				**†Leptin**			
LCA Model	**β Coefficient**	**CI 95%**	***p***	**R^2**	**β Coefficient**	**CI 95%**	***p***	**R^2**	**β Coefficient**	**CI 95%**	***p-value***	**R^2**
#Active & Sedentary LS + Inactive & Non-Sedentary LS	1	-	**-**	-	-	-	-	-	-	-	-	-
Inactive & Sedentary LS	0.455	0.047-0.354	**0.01**‡	0.021	-0.113.	0.29-0.03	**0.117**‡	0.008	0.117	.0.086-0.320	0,257	0.004
BF%	0.007	0.001-0.015	**0.085** ‡	0.00	0.001	0.006-0.010	0.588	0.001	0.069	0.062-0.076	**<0,001**‡	0.5
SBP (mmHg)	0.003	0.003-0.009	0.317	0.003	-0.008	0.014-.0.003	**0.004** ‡	0.022	0.023	0.016-0.03	**<0,001**‡	0.108
DBP (mmHg)	0.003	0.005-0.010	0.646	0.001	-0.014	0.021-.0.006	**0.001** ‡	0.03	0.028	0.018-0.037	**<0,001**‡	0.085
Total Cholesterol (mg/dL)	0.00000	0.002-0.003	0.864	0.00	0.00	0.002-0.003	0.814	<0.001	0.002	.0.001-0.005	**0,144** ‡	0.006
HDL (mg/dL)	-0.006	0.012- 0.001	**0.039** ‡	0.011	0.001	0.005-0.007	0.787	<0.001	-0.01	.0.017-.0.137	**0,007** ‡	0.137
LDL (mg/dL)	0	0.003-0.003	0.796	0.00	-0.001	0.003-0.002	0.723	<0.001	0.004	0.001-0.008	**0,022** ‡	0.013
VLDL (mg/dL)	0.014	0.005-0.023	**0.003** ‡	0.023	0.007	0.002-0.017	**0.109** ‡	0.007	0.013	0.002-0.024	**0,026** ‡	0.013
Triglycerides (mg/dL)	0.003	0.001-0.005	**0.003** ‡	0.023	0.001	0.000-0.003	**0.109** ‡	0.007	0.003	0.000-0.005	**0,026** ‡	0.013
Glucose (mg/dL)	0.007	0.001-0.017	**0.093** ‡	0.007	-0.005	0.014-0.004	0.306	0.003	0.002	.0.007-.0.011	0,636	0.001
HOMA-IR	0.027	0.043-0.096	0.452	0.001	0.079	0.010-0,148	**0.026**‡	0.013	0.319	0.239-0.399	**<0,000** ‡	0.138
Uric Acid(mg/dL)	0.011	0.079-0.057	0.746	0.00	0.036	0.032-0.104	0.299	0.003	0.24	0.157-0.324	**<0,001** ‡	0.77
hs-CRP (mg/dL)	0.414	0.122-0.705	**0.005** ‡	0.02	0.146	0.147-0.439	0.327	0.003	0.743	0.386-1.101	**<0,001** ‡	0.042

#class 1 and class 2 collapsed; †Cardiometabolic Markers that showed normal distribution after logarithmic transformation. ‡ Variables with values of p <0.200 that will be considered for the multiple regression model.

LCA: Latent Class Analysis; LS: Lifestyle; BF%: Body Fat Percentage; SBP: Systolic Blood Pressure; DBP: Diastolic Blood Pressure; HDL: High Density Lipoprotein; LDL: Low Density Lipoprotein; VLDL: Very Low Density lipoprotein; HOMA-IR: Homeostasis Model Assessment – Insulin Resistance; hs-CRP: high sensitivity C-reactive Protein.
